# Assessment of Gastric Myoelectrical Activity in Patients With Type 2 Diabetes Mellitus Using Electrogastrography

**DOI:** 10.7759/cureus.112276

**Published:** 2026-07-08

**Authors:** Vivek Bharti, Subodh Pandey, Somnath S Raghuvanshi, Gourav Raghuwanshi

**Affiliations:** 1 Physiology, Gandhi Medical College, Bhopal, IND; 2 General Medicine, Gandhi Medical College, Bhopal, IND

**Keywords:** autonomic neuropathy, diabetic gastroparesis, electrogastrography, gastric dysrhythmia, gastric emptying, gastric motility, gastric slow waves, gastroparesis, glycated hemoglobin, type 2 diabetes mellitus

## Abstract

Background

Gastric motility problems are a commonly overlooked complication of type 2 diabetes mellitus (T2DM). Diabetic autonomic neuropathy and dysfunction of the interstitial cells of Cajal are believed to be the main underlying mechanisms. Electrogastrography (EGG) is a simple, noninvasive method for studying gastric electrical activity. There are limited data on gastric electrical abnormalities in Indian patients with T2DM. This study was designed to estimate the prevalence of such abnormalities and to examine whether they are related to long-term glycemic control and duration of diabetes.

Methods

This cross-sectional observational study was conducted at the Department of Physiology, Gandhi Medical College, Bhopal. A total of 130 adult patients with T2DM were enrolled. Surface EGG was recorded using a BIOPAC MP36 data acquisition system (BIOPAC Systems Inc., Goleta, CA, USA) before and after a standardized test meal. Gastric slow waves were classified as bradygastria (0.5 to <2 cpm), normogastria (2 to 4 cpm), or tachygastria (>4 to 9 cpm) using a delta T-based wave-count method applied over a standardized 22-minute analyzable window. Dominant frequency (DF), dominant power, instability coefficients, and power ratio were also calculated. Abnormal gastric electrical rhythm (AGER) was defined as normogastria of 70% or less, DF outside the 2 to 4 cpm range, or a postprandial PR of 1 or less. Spearman correlation was used to assess associations between EGG parameters and HbA1c and duration of diabetes.

Results

The mean age of participants was 49.91 ± 9.28 years, and the mean HbA1c was 9.92 ± 2.62%. AGER was found in 92.3% of participants before the meal and in 91.5% after the meal. The proportion of normogastria was markedly low (preprandial, 39.5%; postprandial, 46.6%), whereas bradygastria was high (preprandial, 44.4%; postprandial, 38.3%). Despite these rhythm abnormalities, DF remained within the normal range in both states (2.80 and 3.00 cpm). Instability coefficients were elevated (0.57 preprandial and 0.54 postprandial) and were comparable to values reported in diabetic gastroparesis. No significant correlation was found between any EGG parameter and HbA1c or duration of diabetes (all p > 0.05).

Conclusions

Most patients with T2DM in this study showed substantial subclinical gastric myoelectrical dysfunction, even in the absence of GI symptoms. This included markedly reduced normogastria, elevated bradygastria, and unstable pacemaker activity. No significant association was observed between EGG parameters and HbA1c or reported duration of diabetes. These findings raise the possibility that factors other than long-term glycemic control and disease duration may contribute to gastric myoelectrical dysfunction in T2DM. Further controlled longitudinal studies are needed to explore this.

## Introduction

In India, diabetes mellitus (DM) has become a major public health problem. Around 89.8 million adults between 20 and 79 years of age are currently affected [1]. A large proportion of the population is also in the prediabetic stage. About 13.9% have impaired glucose tolerance, and 11.7% have impaired fasting glucose. Both conditions carry a significant risk of progressing to type 2 DM (T2DM). More concerning is that nearly 43.0% of people with diabetes in India, approximately 38.6 million individuals, remain undiagnosed [1]. Because hyperglycemia often goes untreated for years, it eventually leads to serious complications. Chronic elevation of blood glucose damages the nervous system and blood vessels, contributing to both microvascular and macrovascular complications [2,3].

One complication that does not receive sufficient attention is disturbed gastric motility. There is a bidirectional relationship between glycemic control and gastric emptying. Hyperglycemia can delay gastric emptying, while delayed gastric emptying can further worsen glycemic control [4]. Autonomic neuropathy and chronic hyperglycemia are considered major factors responsible for the disruption of normal gastric function in diabetes [4,5]. Increasing evidence suggests that gastric electrical abnormalities may occur even before the development of clinically evident gastroparesis [5,6].

Electrogastrography (EGG) is a noninvasive technique used to record gastric electrical activity through electrodes placed on the abdominal skin [7]. The technique was first described by Alvarez in 1922 [5,8]. EGG records gastric slow waves, which are rhythmic electrical signals generated by the interstitial cells of Cajal (ICC), the pacemaker cells of the stomach [9]. Abnormal gastric electrical activity detected on EGG is believed to reflect underlying disturbances in gastroduodenal physiology [6]. Because it is safe, simple, and relatively inexpensive, EGG has become an important tool in gastric motility research [7].

In patients with T2DM, abnormal gastric electrical patterns are being increasingly reported. Several studies have demonstrated associations between EGG dysrhythmias and glycemic parameters such as fasting blood glucose and HbA1c [5]. Meta-analyses have also shown reduced normogastria and increased bradygastria in patients with gastroparesis compared with healthy controls [10].

However, data regarding subclinical gastric electrical abnormalities among Indian patients with T2DM remain limited. Unlike previous studies that evaluated dominant frequency (DF), percentage normogastria, or power ratio (PR) individually, the present study used a combined assessment of these complementary electrogastrographic parameters to provide a more comprehensive evaluation of gastric electrical rhythm in patients with T2DM.

The primary objective of this study was to determine the prevalence of abnormal gastric electrical rhythm (AGER) in patients with T2DM using EGG. The secondary objective was to assess whether EGG parameters were associated with HbA1c levels and duration of diabetes.

## Materials and methods

Study design and participants

This study was carried out at the Department of Physiology in collaboration with the Endocrinology Clinic, Gandhi Medical College, Bhopal. A total of 130 patients diagnosed with T2DM were enrolled. All participants were 18 years of age or older. The study was approved by the Institutional Ethics Committee, Gandhi Medical College, Bhopal (approval 90/IEC/2024, dated May 6, 2024). Written informed consent was obtained from every participant before enrollment. All EGG recordings, preprocessing, and signal analyses were performed by a single investigator using a standardized protocol to ensure methodological consistency.

The sample size was calculated using the following formula:

$n = \frac{z^{2}p(1-p)}{d^{2}},$

where z = 1.96 (95% confidence level), p = 9.6% (prevalence of T2DM in India, based on published data [11]), and d = 0.05 (desired precision of 5%). This yielded a minimum sample size of 134, which was rounded up to 140. Since EGG-specific prevalence data for Indian patients with T2DM were not available at the time of study design, the diabetes prevalence figure was used as a pragmatic estimate. Of the 140 participants initially screened, 10 were excluded because recent laboratory reports were unavailable. The final study sample comprised 130 participants, all of whom had complete glycemic and EGG data.

Inclusion criteria

Adult patients (≥18 years) with a confirmed diagnosis of T2DM attending the Endocrinology Clinic at Gandhi Medical College, Bhopal, who were willing to participate and underwent EGG after an overnight fast of at least six hours, with restriction of liquids, including water, for at least two hours before the procedure and without taking any medications during the preceding six hours, were included in the study.

Exclusion criteria

Patients were excluded if they were unwilling to provide consent or had a cardiac pacemaker, GI malignancy, gastroesophageal reflux disease, inflammatory bowel disease, pregnancy, or a history of major abdominal surgery.

Clinical evaluation

Each participant was informed about the study procedure in detail. A general physical examination was performed to confirm eligibility. Demographic details, relevant medical history, and anthropometric measurements, including height, weight, and BMI, were recorded. Written informed consent for EGG was then obtained.

Drug History

In our clinic, the most commonly prescribed antidiabetic medications were metformin, glimepiride, gliclazide, pioglitazone, repaglinide, and dapagliflozin. None of the participants were receiving GLP-1 receptor agonists, DPP-4 inhibitors, proton pump inhibitors, or prokinetic agents that could potentially influence gastric motility.

Electrogastrographic recording

EGG was recorded using a BIOPAC MP36 data acquisition system (BIOPAC Systems Inc., Goleta, CA, USA) with Biopac Student Lab Pro software. Participants were asked to lie comfortably in the supine position at room temperature. They were instructed to relax and avoid movement during recording to minimize artifacts [5,6]. EGG signals were recorded at a sampling rate of 10 Hz. A band-pass filter of 0.016-0.3 Hz was used to isolate gastric slow-wave activity. Each recording session lasted 30 minutes [7].

Patient preparation

All participants fasted for at least six hours before the procedure. Liquids, including water, were restricted for at least two hours before recording [4]. Metallic jewelry was removed to avoid electrical interference [5,8]. The abdominal skin was cleaned with spirit swabs to reduce skin impedance and improve electrode contact [4,6].

Electrode placement

Disposable Ag/AgCl surface electrodes were placed on the abdomen [5,6]. The active positive electrode was placed on the midline, halfway between the xiphoid process and the umbilicus [4,9]. The active negative electrode was placed at approximately 45° from the positive electrode along the left midclavicular line [4]. The reference electrode was placed in the left lower quadrant near the left costal margin [4,5].

Test meal

After completion of the fasting recording, participants were provided with a standardized test meal consisting of 38 g of oats (3.3 g protein, 26.6 g carbohydrates, 3.5 g sugars, 3.8 g dietary fiber, 2.9 g fat; total energy, 138 kcal) along with 250 mL of water. Participants were asked to finish the meal within 10 minutes, after which postprandial EGG recording was performed for 30 minutes.

Signal processing

Raw EGG signals were processed using AcqKnowledge software (BIOPAC Systems Inc.). A digital band-pass filter with cutoff frequencies of 0.016 to 0.3 Hz was applied to isolate gastric slow-wave activity [7]. This filter covers the physiological gastric frequency range of approximately 1 to 18 cpm, which includes normogastria (2 to 4 cpm), bradygastria (0.5 to <2 cpm), and tachygastria (>4 to 9 cpm) [4-8]. The upper cutoff of 0.3 Hz helped exclude cardiac and respiratory signal contamination [8].

The Fast Fourier transform was applied to the filtered signal to obtain the frequency spectrum. DF was defined as the frequency with the maximum spectral power within the gastric range [4-6,8]. Power spectral density analysis was performed, and dominant power (DP) was defined as the maximum power at the DF [3,4]. The percentages of normogastria, bradygastria, and tachygastria, along with DF and DP, were calculated separately for preprandial and postprandial recordings.

Following signal processing, the EGG data were exported from AcqKnowledge to Microsoft Excel (Microsoft Corporation, Redmond, WA, USA). The software generated delta T values, which represent the time interval in seconds between successive slow-wave peaks. These were converted to cpm using the following formula:

$\mathrm{CPM} = \frac{60}{\Delta T}$

Each slow wave was then classified as bradygastria (0.5 to <2 cpm), normogastria (2 to 4 cpm), or tachygastria (>4 to 9 cpm) based on its cpm value, using a spreadsheet formula applied uniformly across all recordings. Raw EGG recordings were visually reviewed for artifacts such as movement-related disturbances, abrupt baseline shifts, and signal interruptions. Segments affected by these artifacts were removed before analysis. Because artifact trimming and signal smoothing resulted in minor variations in analyzable recording duration across participants, wave counting and rhythm classification were performed over a standardized 22-minute analyzable window from each 30-minute recording. This approach ensured consistency and comparability of EGG analysis across all participants. The percentage of each rhythm type was calculated as the number of waves in that category divided by the total wave count over the 22-minute window.

Normal gastric electrical rhythm (NGER) and AGER

In the present study, NGER was defined using a combined assessment of established electrogastrographic parameters. Participants were considered to have NGER when all of the following criteria were fulfilled: DF within 2 to 4 cpm, normogastria comprising >70% of the total recording duration [3,7,9], and a postprandial PR >1, reflecting normal augmentation of gastric electrical activity following meal intake [3,7]. PR was calculated as the ratio of postprandial to preprandial DP [6].

Previous studies have used DF, percentage normogastria, or PR individually as markers of normal or abnormal gastric myoelectrical activity [4,5,9]. However, in the present study, these complementary parameters were evaluated together to provide a more comprehensive assessment of gastric electrical rhythm. Participants who failed to meet one or more of these criteria were classified as having AGER.

Statistical analysis

Data were analyzed using the Numiqo online statistics calculator (Numiqo Team, numiqo e.U., Graz, Austria) [12]. All continuous variables were tested for normality. Since the data were nonnormally distributed, nonparametric tests were used throughout. The Shapiro-Wilk test was used to assess normality. The Wilcoxon signed-rank test was used to compare preprandial and postprandial EGG parameters. Spearman rank correlation was used to assess associations between EGG parameters and HbA1c and duration of diabetes. Normally distributed continuous variables are expressed as mean ± SD and nonnormally distributed variables as median with IQR. Categorical variables are expressed as frequencies and percentages. A p-value <0.05 was considered statistically significant. Since multiple EGG parameters were tested, these findings should be interpreted as exploratory.

## Results

Demographic and clinical characteristics

A total of 130 patients were studied. The mean age was 49.91 ± 9.28 years. The mean body weight was 67.64 ± 11.10 kg, and the mean height was 161.20 ± 8.56 cm, giving a mean BMI of 25.92 ± 2.93 kg/m². The mean systolic blood pressure was 129.28 ± 4.46 mmHg, and the mean diastolic blood pressure was 84.28 ± 4.39 mmHg.

Glycemic profile

Glycemic values were elevated across all parameters. The mean RBS was 221.03 ± 75.18 mg/dL, the mean PPBS was 261.25 ± 87.48 mg/dL, and the mean FBS was 173.69 ± 65.77 mg/dL. The mean HbA1c was 9.92 ± 2.62%, indicating poor long-term glycemic control in most participants. The mean duration of diabetes was 5.72 ± 4.53 years.

Gastric electrical rhythm classification

**Figure 1:**
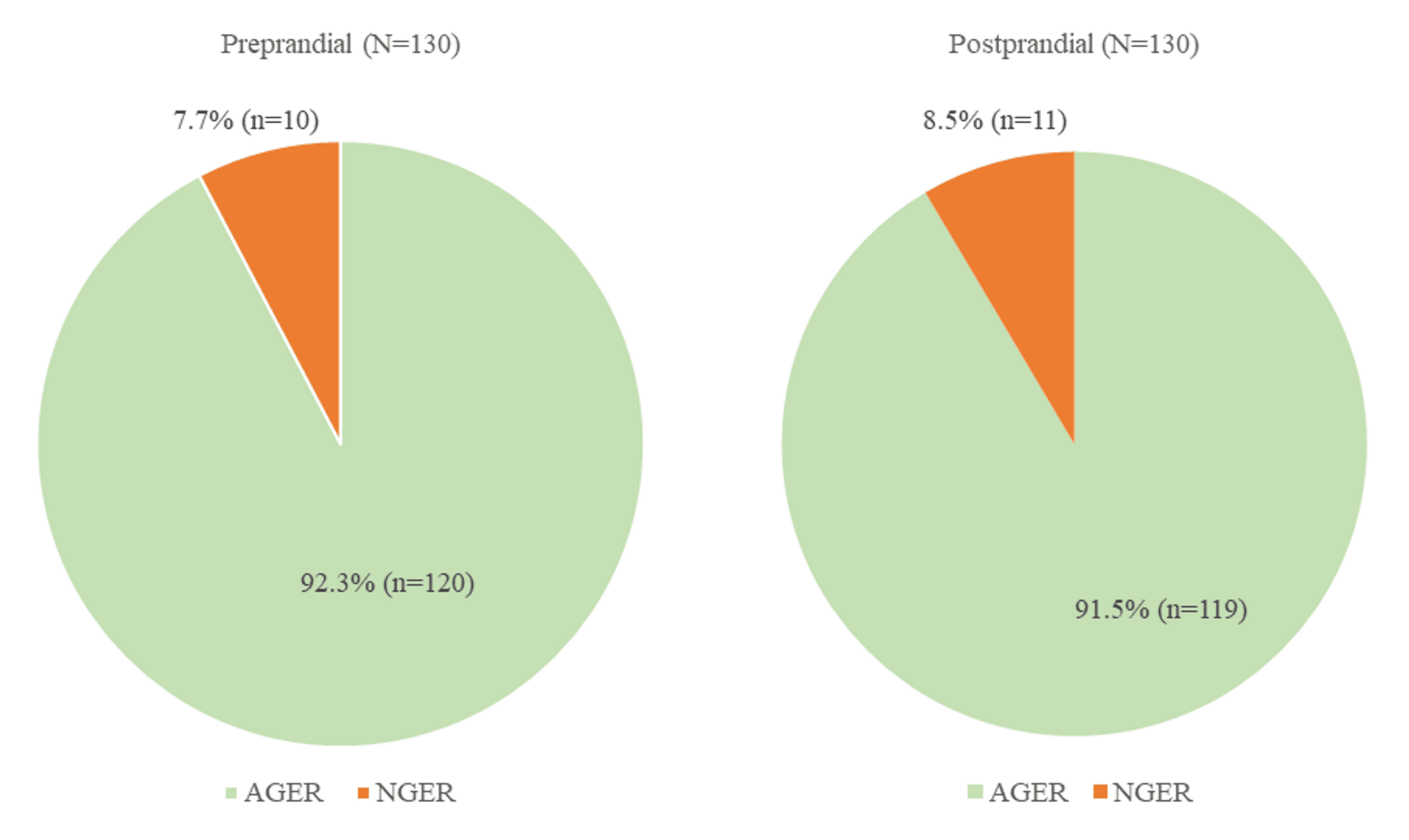
Gastric electrical rhythm classification in T2DM patients (N = 130) N represents the total study population, whereas n represents the number of participants within each subgroup. AGER, abnormal gastric electrical rhythm; NGER, normal gastric electrical rhythm; T2DM, type 2 diabetes mellitus

As shown in Figure 1, in the preprandial state, AGER was observed in 120 participants (92.3%). Only 10 participants (7.7%) had NGER. After the meal, the proportion with NGER increased marginally to 11 participants (8.5%), whereas AGER persisted in 119 participants (91.5%). Only one participant had NGER in both the preprandial and postprandial states. These findings indicate that postprandial normalization of gastric electrical activity was minimal in this cohort.

GI symptoms

Table 1 summarizes the GI symptoms reported by the participants. Of the 130 individuals included in the study, 54 (41.5%) did not report any symptoms, whereas 76 (58.5%) experienced at least one GI complaint. The most frequently reported symptom was early satiety, affecting 52 participants (40.0%). Reduced appetite was reported by 28 participants (21.5%), and postprandial fullness was reported by 22 participants (16.9%). Some participants experienced more than one symptom simultaneously. Overall, the pattern of symptoms was similar in male and female participants. GI symptoms were summarized using descriptive statistics.

**Table 1:** Distribution of GI symptoms among study participants Data are presented as the number of participants (n) and percentage (%). N represents the total study population. Several participants reported more than one symptom simultaneously.

Symptom category	Male (n = 74)	Female (n = 56)	Total (N = 130)	Percentage (%)
No symptoms	30	24	54	41.5
Early satiety	28	24	52	40.0
Postprandial fullness	14	8	22	16.9
Bloating	8	10	18	13.8
Decreased appetite	16	12	28	21.5
Constipation	10	7	17	13.1
Increased appetite	4	2	6	4.6

Comparison of preprandial and postprandial EGG parameters

Table 2 shows the preprandial and postprandial EGG parameters. Significant postprandial changes were observed in DF, DP, normogastria, bradygastria, and the instability coefficient of dominant power (IC-DP; all p < 0.05). Tachygastria and IC-DF did not change significantly after the meal.

**Table 2:** Comparison of preprandial and postprandial EGG parameters in T2DM patients (N = 130) Data are expressed as median (Q1-Q3). p-values were obtained using the Wilcoxon signed-rank test. Statistical significance was defined as p < 0.05. DF, dominant frequency; DP, dominant power; EGG, electrogastrography; IC-DF, instability coefficient of dominant frequency; IC-DP, instability coefficient of dominant power; T2DM, type 2 diabetes mellitus

EGG parameter	Preprandial median (Q1-Q3)	Postprandial median (Q1-Q3)	p-value	95% CI (pre-post)
DF (cpm)	2.8 (2.13-3.00)	3.0 (2.50-3.30)	<0.001	-0.30 to -0.15
DP (mV)²/Hz)	0.001 (0.0004-0.004)	0.003 (0.0009-0.008)	<0.001	-0.00235 to -0.00100
Normogastria (%)	39.5 (28.05-52.15)	46.6 (30.23-58.18)	<0.001	-7.93 to -3.30
Bradygastria (%)	44.4 (28.43-60.60)	38.3 (25.00-54.05)	<0.001	2.61 to 7.98
Tachygastria (%)	12.0 (5.38-17.20)	12.35 (6.42-18.64)	0.42	-1.705 to 1.20
IC-DF	0.57 (0.47-0.72)	0.54 (0.45-0.64)	0.05	-0.01 to 0.07
IC-DP	0.31 (0.22-0.45)	0.34 (0.25-0.46)	0.003	-0.045 to -0.010

**Figure 2:**
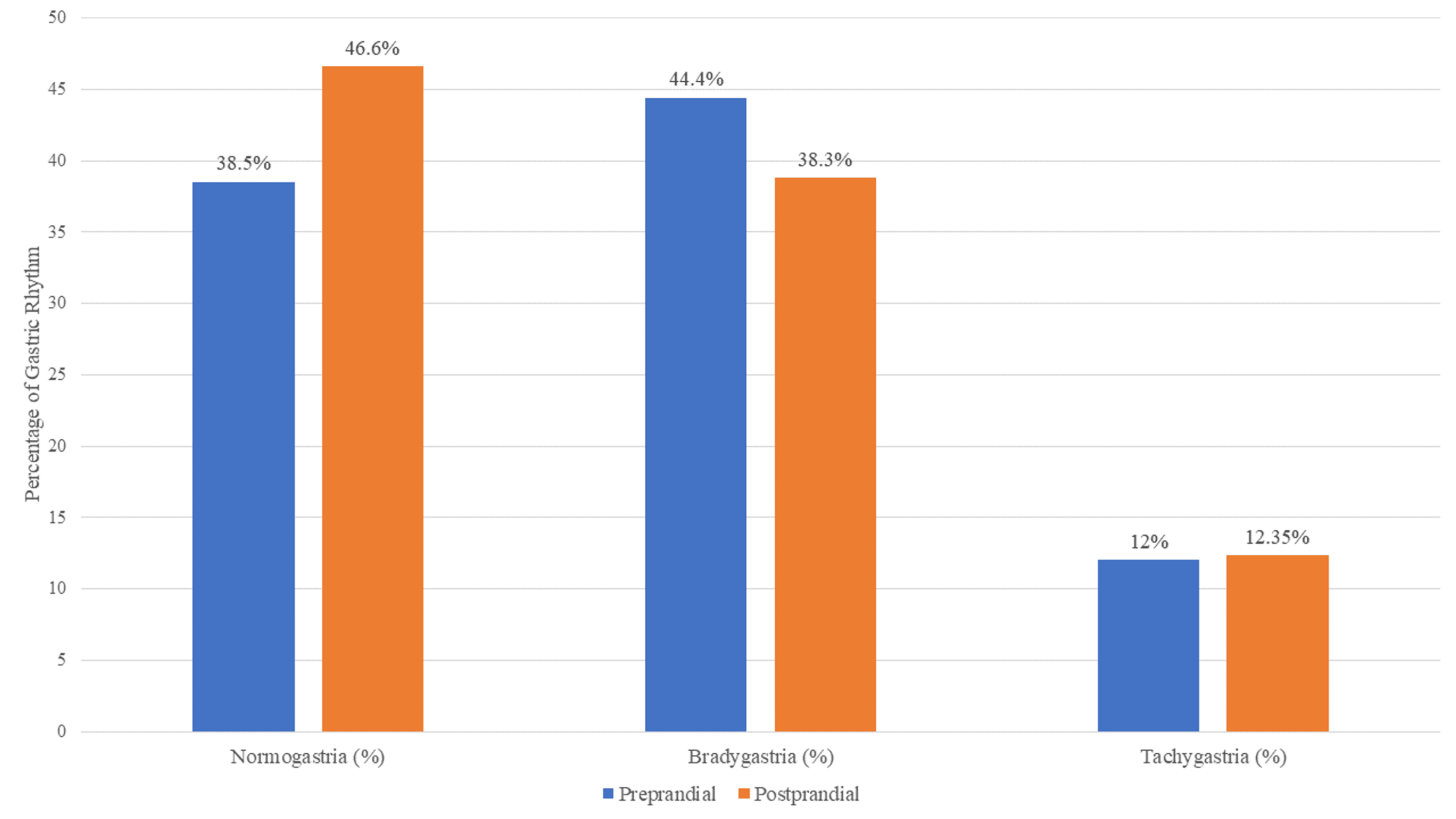
Preprandial and postprandial gastric rhythm distribution in T2DM patients (N = 130) Data are expressed as the median percentage (%) of normogastria, bradygastria, and tachygastria across the study participants. T2DM, type 2 diabetes mellitus

Figure 2 shows the preprandial and postprandial gastric rhythm distribution. 

Table 3 presents the results of the Spearman correlation analysis. No statistically significant correlation was found between any EGG parameter and either HbA1c or duration of diabetes. This finding was consistent for both the preprandial and postprandial values of DF, DP, normogastria, bradygastria, tachygastria, and PR (all p > 0.05).

**Table 3:** Spearman rank correlation of EGG parameters with HbA1c and duration of diabetes (N = 130) Correlation analysis was performed using the Spearman rank correlation test. Statistical significance was defined as p < 0.05. None of the correlations were statistically significant. DF, dominant frequency (mHz); DP, dominant power (mV²/Hz); EGG, electrogastrography; PR, power ratio; rho, Spearman rank correlation coefficient

EGG parameter	Duration of diabetes (rho)	Duration of diabetes (p-value)	HbA1c (rho)	HbA1c (p-value)
Preprandial DF	0.055	0.536	0.162	0.066
Postprandial DF	0.04	0.656	0.102	0.248
Preprandial DP	-0.015	0.862	0.119	0.177
Postprandial DP	-0.003	0.97	0.103	0.246
Preprandial normogastria (%)	0.056	0.53	0.036	0.688
Postprandial normogastria (%)	-0.016	0.857	0.11	0.212
Preprandial tachygastria (%)	-0.019	0.834	0.014	0.871
Postprandial tachygastria (%)	0.083	0.35	-0.066	0.453
Preprandial bradygastria (%)	-0.03	0.733	0.001	0.995
Postprandial bradygastria (%)	-0.005	0.958	-0.074	0.406
PR	-0.011	0.901	0.016	0.858

## Discussion

Gastric rhythm analysis

To put the findings of this study into context, it is helpful to consider what has been reported in healthy individuals. Fasting normogastria of 80.38% (95% CI: 76.88-84.04%) and postprandial normogastria of 83.65% have been reported in healthy individuals [10], with bradygastria of 9.08% preprandially and 7.70% postprandially, and tachygastria of 6.00% preprandially and 4.87% postprandially [10]. In another study, preprandial normogastria of 91.6% and postprandial normogastria of 83.3% were reported in healthy controls, with preprandial bradygastria and tachygastria of 3.5% each [5]. Mean preprandial and postprandial normogastria of 87.5% and 90.4%, respectively, have also been reported [6]. In patients with T2DM, preprandial and postprandial normogastria of 54.1% and 48.9%, bradygastria of 20.4% and 24.5%, and tachygastria of 17.6% and 22.2%, respectively, have been documented [3]. The values observed in the present study were in the same direction but more pronounced (Figure 2).

In this cohort, normogastria was only 39.5% preprandially and 46.6% postprandially. Bradygastria was 44.4% preprandially and 38.3% postprandially. Tachygastria was 12.0% preprandially and 12.35% postprandially. Although normogastria and bradygastria showed statistically significant improvement after the meal, the changes were modest and remained well below normal values. Tachygastria did not change significantly after eating (p = 0.42), indicating no postprandial modulation of high-frequency rhythm in this group.

Taken together, these findings point to substantial impairment of gastric electrical regulation in T2DM. Under normal conditions, eating activates vagal and hormonal pathways that bring the gastric slow-wave frequency toward 3 cpm, reduce dysrhythmias, and coordinate gastric contractions [3,4,13]. In the present cohort, these normal postprandial physiological responses appeared to be impaired. The most likely explanation is damage to the autonomic nerve supply of the stomach, reduced vagal tone, and loss of ICC function, all of which disrupt the organized electrical activity required for effective gastric motility [13,14].

One notable finding was that 41.5% of participants were completely asymptomatic despite significant AGER on EGG. This finding is clinically relevant because it suggests that a substantial proportion of patients with T2DM have significant gastric electrical dysfunction without recognizable symptoms. Symptom-based screening alone would therefore fail to identify many of these patients.

DF

The DF increased from 2.80 cpm (2.13-3.00) preprandially to 3.00 cpm (2.50-3.30) postprandially. This change was statistically significant (p < 0.001). Both values remained within the normal gastric range of 2 to 4 cpm [9]. Similar DF values have been reported in healthy individuals. Median fasting and postprandial DF values of 2.90 and 3.03 cpm, respectively, have been documented [10]. Mean preprandial and postprandial DF values of 2.7 ± 0.2 cpm and 2.9 ± 0.2 cpm, respectively, have also been reported [6]. Median preprandial and postprandial DF values of 2.8 and 3.0 cpm, respectively, have likewise been observed [5].

Thus, the DF observed in patients with T2DM in the present study appears similar to that reported in healthy individuals. However, DF alone may not adequately reflect overall gastric electrical stability. A normal DF does not necessarily indicate normal gastric electrical activity because it represents only the frequency at which maximum spectral power occurs. It provides no information about the proportion of time during which the stomach maintains a normal rhythm [7,8]. Even when slow waves are generated at a normal frequency, their propagation and stability may be disrupted by conduction abnormalities, ectopic pacemaker activity, or spatial dysrhythmias [15]. In T2DM, diabetic autonomic neuropathy (DAN) and ICC damage are likely contributors [3]. The markedly reduced normogastria observed in the present study despite a normal DF illustrates this dissociation.

Instability of gastric electrical rhythm

The instability of the gastric pacemaker was assessed using IC-DF and IC-DP. IC-DF was 0.57 (IQR: 0.47-0.72) preprandially and 0.54 (IQR: 0.46-0.66) postprandially. IC-DP was 0.31 (IQR: 0.22-0.45) preprandially and 0.34 (IQR: 0.25-0.47) postprandially. These values indicate substantial variability in both the frequency and power of gastric electrical activity.

The instability coefficient measures how much the DF varies over time. Lower values indicate a stable, well-coordinated rhythm, whereas higher values indicate an irregular and dysrhythmic rhythm [7,9]. In healthy individuals, IC-DF is low. A preprandial IC-DF of 0.37 ± 0.24 has been reported in healthy controls, compared with 0.60 ± 0.19 preprandially and 0.57 ± 0.21 postprandially in patients with diabetic gastroparesis [15]. The values in the present study (0.57 preprandially and 0.54 postprandially) are very close to those reported in diabetic gastroparesis. This suggests a similar degree of pacemaker dysfunction even in patients with T2DM without confirmed gastroparesis.

These findings also explain the apparent contradiction observed with DF. Although DF remained within the normal range, the elevated IC-DF indicates that this frequency was not maintained consistently [8,9]. Frequent fluctuations between normal and abnormal rhythm patterns may have occurred. This likely explains why normogastria was only 39.5% preprandially despite a normal DF. Residual ICC may still generate slow waves at near-normal frequencies, but the overall pacemaker network may be disrupted by DAN and microvascular changes, resulting in rhythm instability and poor electromechanical coupling [3,4].

PR

When participants were categorized according to PR, 116 (89.2%) had a PR of ≥1, whereas 14 (10.8%) had a PR of <1. Among those with a PR of <1, the mean was 0.55 ± 0.29, and the median was 0.60 (0.28-0.80). Among those with a PR of ≥1, the mean was 2.26 ± 1.30, and the median was 2.0 (1.2-2.7).

PR is the ratio of postprandial to preprandial DP [9]. In healthy individuals, a PR >1 reflects normal postprandial augmentation of gastric electrical activity, which requires intact vagal reflexes and preserved gastric contractile function [9]. The 10.8% of participants with a PR <1 showed a blunted postprandial response. This finding is generally associated with delayed gastric emptying, advanced DAN, and ICC dysfunction in T2DM [3,13].

The more notable finding was that 89.2% of participants had a PR of ≥1 despite a postprandial normogastria of only 46.6%. This suggests that although the electrical activity increased in amplitude after the meal, it remained poorly coordinated. Two mechanisms may explain this observation. First, gastric distension after eating reduces the distance between the stomach and the surface electrodes, which can artificially increase signal amplitude and produce a falsely elevated PR even when gastric contractions are weak [3,9]. Second, EGG reflects total gastric electrical activity rather than coordinated contractions alone [9]. In T2DM, meal ingestion may trigger disorganized patterns of bradygastria and tachygastria that increase signal amplitude without producing effective gastric emptying [3,7].

Thus, a PR >1 in most participants should not be interpreted as evidence of normal gastric function. Rather, it indicates increased electrical amplitude without corresponding improvement in coordination, which is consistent with underlying DAN and ICC dysfunction.

Relationship with HbA1c and duration of diabetes

No statistically significant correlation was found between HbA1c and any of the EGG parameters, including normogastria, bradygastria, tachygastria, DF, and PR, in either the preprandial or postprandial state (p > 0.05) [3]. Similarly, no significant correlation was found between the duration of diabetes and any EGG parameter [3]. These findings suggest that gastric myoelectrical abnormalities may not be closely related to long-term glycemic control or duration of diabetes. However, longitudinal studies are needed to better understand these relationships. It is possible that factors such as DAN and dysfunction of the ICC contribute to these abnormalities, although this hypothesis requires confirmation in prospective studies.

This is an important finding from a pathophysiological perspective. Once the gastric neuromuscular apparatus has undergone structural damage due to DAN and ICC loss, the resulting electrical instability may persist regardless of current glycemic control or disease duration [3,4]. Improving glycemic control may not reverse this damage once it has occurred.

Clinical implications and dietary management

The EGG findings showed a modest but significant postprandial increase in normogastria and a reduction in bradygastria. This suggests that the gastric pacemaker, although substantially impaired, still retains some responsiveness to a small meal. This observation is consistent with the rationale underlying dietary advice commonly provided for diabetic gastric dysfunction, such as consuming small, frequent meals and avoiding high-fat and high-fiber foods [2]. Prospective studies that evaluate dietary interventions alongside gastric emptying measurements are needed to determine whether these strategies improve EGG parameters.

Limitations

This study has several limitations. A matched healthy control group was not included; therefore, EGG values were compared with published normative data from previous studies. Differences in ethnicity, methodology, and analytical techniques across studies may limit direct comparisons. Gastric rhythm was assessed using a delta T-based wave-count method rather than power spectral density analysis, which may limit comparability with other EGG studies. As this was a cross-sectional study, causal or temporal relationships between T2DM and gastric myoelectrical abnormalities could not be established. Detailed information regarding medication dosage, duration, and combination therapy was not systematically collected. The most commonly prescribed antidiabetic medications in our clinic were metformin, glimepiride, gliclazide, pioglitazone, repaglinide, and dapagliflozin, and none of the participants were receiving GLP-1 receptor agonists. However, the potential influence of other medications on the EGG findings cannot be completely excluded. Sex-disaggregated analysis of EGG parameters was not performed because of the exploratory nature of the study. Future studies should include matched healthy Indian controls, use power spectral density-based rhythm analysis, examine sex-based differences in gastric myoelectrical activity, and use validated symptom assessment tools such as the GCSI to investigate the relationship between EGG findings and symptom severity.

## Conclusions

This study found that abnormal gastric myoelectrical rhythm was present in more than 90% of the patients with T2DM. The main features were reduced normogastria, elevated bradygastria, and unstable pacemaker activity. Instability coefficients were comparable to those reported in diabetic gastroparesis. A substantial proportion of participants were completely asymptomatic, indicating that symptom-based assessment alone is insufficient to detect gastric dysfunction in T2DM. No significant correlation was found between EGG parameters and HbA1c or duration of diabetes. Larger prospective studies incorporating detailed assessment of autonomic neuropathy, medication use, and gastric emptying are required to further investigate these relationships. These findings raise the possibility that factors beyond glycemic control and disease duration contribute to gastric myoelectrical dysfunction. EGG appears to be a useful noninvasive tool for detecting subclinical gastric myoelectrical dysfunction in T2DM, but controlled studies including matched healthy controls and gastric emptying data are needed before any screening recommendations can be made. The modest postprandial improvement in normogastria and bradygastria suggests that small, frequent meals may help reduce GI symptoms in this population.
